# Impostorism in third-year medical students: an item analysis using the Clance impostor phenomenon scale

**DOI:** 10.1007/s40037-020-00562-8

**Published:** 2020-02-06

**Authors:** Beth Levant, Jennifer A. Villwock, Ann M. Manzardo

**Affiliations:** 1 Department of Pharmacology, Toxicology, and Therapeutics, University of Kansas Medical Center, Kansas City, KS USA; 2 Department of Otolaryngology, University of Kansas Medical Center, Kansas City, KS USA; 3 Department of Psychiatry and Behavioral Sciences, University of Kansas Medical Center, Kansas City, KS USA

**Keywords:** Impostor, Medical student, Gender, Burnout

## Abstract

**Introduction:**

Impostorism, feelings of distrust in one’s abilities and accomplishments despite evidence to the contrary, is frequent in medical students and negatively affects student wellness.

**Methods:**

The aspects of impostorism that were most prevalent in medical students during the transition from the preclinical to clinical phases of their training were assessed using an anonymous, voluntary 60-item survey that included the Clance Impostor Phenomenon Scale (CIPS) and a 2-item burnout assessment administered in October-November 2018. Ratings of individual CIPS items were compared between items for the entire sample and in subpopulations of students. The correlation of individual CIPS items with CIPS total score was also determined.

**Results:**

A total of 127 of 215 (59%) surveyed students responded, with 112 completing the CIPS with mean score of 63.0 ± 14.6 (moderate-to-frequent impostor feelings). Ratings of individual CIPS items differed significantly between items. Responses also differed depending on gender and perceived burnout or depersonalization.

**Discussion:**

Third-year medical students identified most strongly with items related to unfounded fear of failure, hesitance to share recognition before it is announced, remembering failures rather than successes, believing themselves less capable than others, and worrying about succeeding. In contrast, attribution of accomplishments to luck was not prominent for these students. Responses to certain items also differed depending on gender and perceived burnout or depersonalization, but not self-reported under-represented minority status. This observation may inform the development of interventions tailored to foster wellness as students negotiate the transition from the preclinical to clinical phases of their training.

**Electronic supplementary material:**

The online version of this article (10.1007/s40037-020-00562-8) contains supplementary material, which is available to authorized users.

## Background

Impostorism is a phenomenon in which individuals distrust their abilities and accomplishments despite evidence of success and competence, and fear they may be exposed as an “impostor” [1, 2]. Although first described in women and thought to be a static trait, impostorism has been shown to occur in both genders and is currently viewed as an evoked affective response to specific situations [3]. The phenomenon has been associated with psychological attributes such as perfectionism, anxiety, and neuroticism; and lower self-discipline, perceived competence, conscientiousness, and resilience [4–8]. It is observed in a variety of populations including ethnic minorities, workers, and university faculty [6, 9, 10].

In medical students and residents, impostor phenomenon occurs in nearly half of females and one-fourth of males, with the intensity of impostor feelings varying across the course of medical training [11–16]. Career transitions, such as beginning a career or moving between phases of a career, are times when impostor feelings are particularly likely to occur [17, 18]. For medical students, the move from the preclinical to the clinical phases of training can be particularly challenging [19–21]. Having frequent impostor feelings has a negative effect on the wellness of medical students, who are noted for having higher prevalence and levels of psychological distress than age-matched peers, manifested as depression, anxiety, and thoughts of dropping out of medical school or suicide, presumably resulting from factors including academic pressure, financial issues, lack of emotional support, and the perception of unattainable expectations [11, 22–26]. Impostorism has also been shown to contribute to burnout and to be an impediment to identity formation as physicians, which in turn can negatively affect patient care [11, 12, 27–29]. There is thus considerable interest in, and efforts directed towards, promoting student wellbeing in medical education, including mitigating impostor feelings [30, 31].

Studies of the Clance Impostor Phenomenon Scale (CIPS) suggest that impostorism may consist of subcategories of beliefs. Two studies in undergraduates suggested that CIPS items can be grouped into three factors that relate to self-doubt and concerns about ability (fake), the attribution of successes to luck (luck), and the inability to internalize success and praise (discount) [32–34]. Another study in undergraduates indicated clustering of CIPS items into four subgroups: fake, luck, discount, and a fourth consisting of item 1, which concerns having succeeded at a task when one was afraid of failure [35]. In contrast, a study in doctoral students in science, technology, engineering, and math fields found a single factor model of impostorism to be most parsimonious [36]. Although the existence of subcategories of impostorism within the CIPS is debatable and may vary depending on the study population, these studies raise the question of whether individual impostors manifest all aspects of impostorism equally, or if certain impostors predominantly express, or do not express, certain impostor beliefs. Understanding how particular types of respondents (i.e., high achievers in demanding fields, such as medical students) are experiencing impostorism would inform the development of preventative and mitigating interventions tailored specifically to target the types of impostor feelings that were being experienced.

Previous studies have examined the incidence and severity of impostorism, its causes and effects, the attributes of impostors, and the efficacy of interventions. As a complement to those studies, this study assessed the aspects of impostorism most strongly manifested in medical students at a time point when they are particularly likely to be experiencing impostor feelings—the early clinical phase of their training—by examining the strength of their ratings of individual CIPS items. We show that medical students’ identification with specific CIPS items differed significantly between items. Items related to unfounded fear of failure, hesitance to share recognition before it is announced, remembering failures rather than successes, believing themselves less capable than others, and unfounded worrying about succeeding were the most strongly rated. In contrast, identification with items related to doubting past accomplishments or attributing successes to luck was not prominent for these students. Furthermore, certain CIPS items appear to be potentially strong indicators of distress in medical students.

## Methods

This study was approved by the University of Kansas Medical Center IRB (STUDY #00142155) and conducted in accordance with the Declaration of Helsinki. Informed consent was obtained from all participants.

### Participants

Third-year medical students (*n* = 215) from the University of Kansas School of Medicine class of 2020 participated in this study. These students were trained in a traditional 4‑year program in which students undergo 2 years of preclinical training, consisting of a lecture-based integrated basic science curriculum with some clinical skills experiences, followed by 2 years of clinical training. Responding students were located on two campuses (65% and 35% of respondents, respectively).

### Data collection

A voluntary, anonymous, 60-item survey was administered in October-November of the 2018 Fall semester. This time-point for data collection was selected so that students would have completed one rotation, but still be in the early phase of their clinical training. The survey consisted of the instruments described below, as well as the Perceived Stress Scale (10 items), which was not used in this analysis. The survey also contained 28 demographic items (e.g., age, race, gender, campus, grade point average, etc.) (see Tab. 1 of the online Electronic Supplementary Material). The large number of demographic items was necessitated by the anonymous design of this study. The preponderance of the demographic data was not used in this analysis.

Data were collected and managed using the REDCap (Research Electronic Data Capture) electronic data capture tool hosted at the University of Kansas Medical Center [37]. The survey required roughly 20 min. to complete. Participation was incentivized by a contribution to the class fund if a specified response rate was achieved.

### Instruments

Impostorism was measured using the Clance Impostor Phenomenon Scale (CIPS) [1] (used with permission). The CIPS is a 20-item survey in which respondents rate their answers on a Likert scale from 1 to 5 for not at all true, rarely true, sometimes true, often true, or very true, respectively. Responses to each item were added to yield a total score ranging from 20 to 100. The higher the score, the more frequently and seriously impostorism interferes in a person’s life. If the total score was 40 or less, the respondent was considered to have few impostor characteristics; 41–60, moderate impostor experiences; 61–80 frequent impostor feelings; and greater than 80, intense impostor experiences [1]. A score of 62 or greater was interpreted as indicating an individual with impostor phenomenon. This cutoff score was based on the observation of one false positive and no false negatives in a sample of 64 subjects assessed for impostor phenomenon by clinical interview [33]. The CIPS has high internal reliability with Cronbach’s α = 0.92 [35], 0.96 [33], and 0.87–0.89 [34]. The correlation of individual CIPS items with the mean total score was 0.47–0.55 [33, 34]. Impostor score was related to, but discriminable from, measures of self-esteem, depression, social anxiety, and self-monitoring, and was strongly correlated with scores on the Perceived Fraudulence Scale [38] (*r* = 0.79, *p* < 0.01) [35]. The CIPS also exhibited superior sensitivity and reliability compared with the Harvey IP Scale [33].

Burnout was assessed using a 2-item instrument developed for and validated in medical professionals by West et al. [39, 40] (licensed from Mind Garden). The instrument consists of 2 single yes/no items of burnout and depersonalization. A positive response to either item was considered to indicate burnout or depersonalization, respectively. These items were derived from the full Maslach Burnout Inventory (MBI) [41]. When assessed in medical professionals, responses to the single-item burnout and depersonalization questions were strongly correlated with the emotional exhaustion and depersonalization domain scores (minus the single-item questions) of the full MBI (Spearman *r* = 0.76–0.83 and 0.61–0.72, respectively) [39]. The positive predictive values of the single-item burnout and depersonalization questions were 88.2% and 89.6% and the positive likelihood ratios were 14.9 and 23.4, respectively [39, 42]. Validation of the emotional exhaustion and depersonalization subscales of the full MIB indicate high and moderate internal reliability (α = 0.89 and 0.67), respectively [43].

### Data analysis

Descriptive statistics (sample means, medians with standard deviations) were generated using SAS statistical software, version 9.4. Eight respondents omitted a response to one item on the CIPS (a different item for each respondent). The total impostor score for these 8 individuals was calculated by multiplying their score from the 19 completed items by 1.05263 and rounding to the nearest whole number.

The scores for individual CIPS items were compared between items by Kruskal-Wallis one-way ANOVA on ranks followed by Dunn’s test. The effects of gender, minority status, and burnout/depersonalization on the distribution of responses to each CIPS item were assessed as planned comparisons using the chi-square test, followed by the Mantel-Haenszel chi-square test. The effects of burnout and depersonalization were analyzed separately. Effect sizes were computed using Cohen’s w for chi-square [44]. The relationships between responses to individual CIPS items and the total CIPS score were determined by Spearman correlation (Instat, 3, GraphPad).

## Results

A total of 127 of 215 (59%) students surveyed responded with 112 completing at least 95% of the CIPS. The mean age of respondents was 25.8 ± 3 years old (range 23–44). Twenty-three percent self-identified as an under-represented minority.

### Between-item comparison of CIPS item responses in the total sample

The mean CIPS score for the entire sample was 63.1 ± 14.6 (moderate-to-frequent impostor feelings) with 41% reporting moderate impostor feelings (score 40–60), 38% frequent impostor feelings (score 60–80), and 13% intense impostor feelings (score >80). CIPS scores were similar for students on both campuses (63.3 ± 15.4 and 62.9 ± 13.3, respectively; *p* > 0.999) and were thus pooled for these analyses.

Comparison of individual items on the CIPS indicated that students’ ratings of items varied significantly (*p* < 0.0001) (Tab. 1). Ratings of five items (1, 7, 17, 18, and 19), were significantly stronger than other CIPS items (*p* < 0.05). These items concerned having succeeded on tasks when one was afraid of failure, hesitance to share recognition before it is announced, remembering failures, believing themselves less capable than others, and unfounded worrying about succeeding, respectively. In contrast, rating of item 9, which describes a lack of confidence in past successes, was notably low and significantly different from responses to items ranked 1‑12 (*p* < 0.05).

**Table 1:** Medical student responses to individual items on the Clance Impostor Phenomenon Scale^a^

Item rank			Clance Impostor Phenomenon Scale Item	Total sample score	Item-total corr. (r)
Total sample	Males	Females			
1	1	3	1. I have often succeeded on a test or task even though I was afraid that I would not do well before I undertook the task	3.91 ± 0.75**	0.12
2	3	2	19. If I’m going to receive a promotion or gain recognition of some kind, I hesitate to tell others until it is an accomplished fact	3.91 ± 1.14***	0.41*******
3	2	4	7. I tend to remember the incidents in which I have not done my best more than those times I have done my best	3.83 ± 1.06****	0.61*******
4	5	1*	17. I often compare my ability to those around me and think they may be more intelligent than I am	3.81 ± 1.13****	0.66*******
5	8	5*	18. I often worry about not succeeding with a project or examination, even though others around me have considerable confidence that I will do well	3.42 ± 1.17*****	0.71*******
6	7	6	16. If I receive a great deal of praise and recognition for something I’ve accomplished, I tend to discount the importance of what I’ve done	3.31 ± 1.23******	0.71*******
7	6	7	12. I’m disappointed at times in my present accomplishments and think I should have accomplished much more	3.28 ± 1.18******	0.64*******
8	9	8	20. I feel bad and discouraged if I’m not “the best” or at least “very special” in situations that involve achievement	3.24 ± 1.22******	0.45*******
9	4	11	2. I can give the impression that I’m more competent than I really am	3.23 ± 1.09******	0.13
10	14	9	14. I’m often afraid that I may fail at a new assignment or undertaking even though I generally do well at what I attempt	3.12 ± 1.10******	0.80*******
11	12	10	13. Sometimes I’m afraid others will discover how much knowledge or ability I really lack	3.09 ± 1.26******	0.79*******
12	11	12	8. I rarely do a project or task as well as I’d like to do it	2.98 ± 1.00******	0.60*******
13	13	16	5. I sometimes think I obtained my present position or gained my present success because I happened to be in the right place at the right time or knew the right people	2.90 ± 1.27	0.59*******
14	10	17	6. I’m afraid people important to me may find out that I’m not as capable as they think I am	2.90 ± 1.24	0.77*******
15	16	13	10. It’s hard for me to accept compliments or praise about my intelligence or accomplishments	2.87 ± 1.26	0.71*******
16	17	15	15. When I’ve succeeded at something and received recognition for my accomplishments, I have doubts that I can keep repeating that success	2.81 ± 1.22	0.80*******
17	15	19	11. At times, I feel my success has been due to some kind of luck	2.79 ± 1.20	0.72*******
18	18	16	3. I avoid evaluations if possible and have a dread of others evaluating me	2.79 ± 1.21	0.50*******
19	19	14	4. When people praise me for something I’ve accomplished, I’m afraid I won’t be able to live up to their expectations of me in the future	2.73 ± 1.25	0.72*******
20	20	20	9. Sometimes I feel or believe that my success in my life or in my job has been the result of some kind of error	2.26 ± 1.22	0.80*******

Data are the mean ± SD (*N* = 111–112).

*p < 0.01 v. males by Chi-square and Mantel-Haenszel Chi-square tests

**p < 0.05 v. Ranks 6–20 for total sample by Kruskal-Wallis one-way ANOVA on ranks followed by Dunn’s test.

***p < 0.05 v. Ranks 7–20 for total sample.

****p < 0.05 v. Ranks 10–20 for total sample.

*****p < 0.05 v. Ranks 17–20 for total sample.

******p < 0.05 v. Rank 20 for total sample.

*******p < 0.0001 by Spearman correlation.

^a^Used and reproduced with permission. From [45]. Copyright 1985 by Pauline Rose Clance, Ph.D., ABPP. Do not reproduce without permission from Pauline Rose Clance, drpaulinerose@comcast.net, www.paulineroseclance.com

### Between-item comparison of CIPS item responses in subgroups of students

**Fig. 1:**
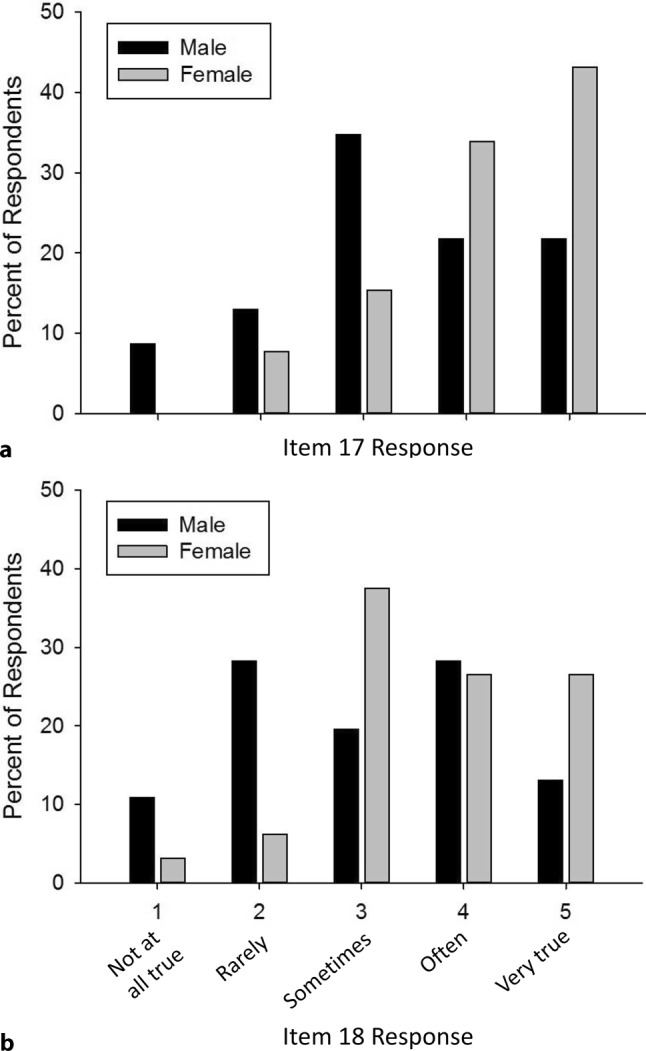
Distributions of responses by male and female medical students to CIPS items 17 (**a**) and 18 (**b**). For item 17, comparing self to others, the mean score (± SD) for males (*n* = 46) was 3.35 ± 1.22; females (*n* = 65), 4.14 ± 0.94. For item 18, worrying about succeding despite the confidence of others, the mean score for males (*n* = 46) was 3.04 ± 1.25; females (*n* = 64), 3.69 ± 1.05. For both items, rating was higher for females (*p* < 0.01) by chi-square and Mantel-Haenszel chi-square tests

Examination of the distribution of responses to individual questions revealed differences in CIPS item ratings based on gender, burnout, and depersonalization. Analysis of the effects of gender indicated differences in the rating of two items, 17 and 18, both of which were more strongly endorsed by females than males (Fig. 1). For item 17 (believing themselves less capable than others), the mean score for males was 3.35 ± 1.22; females, 4.14 ± 0.94 (*p* < 0.01, effect size = 0.38). Item 17 was the top ranked CIPS item for females. For item 18 (unfounded worrying about succeeding), the mean score for males was 3.04 ± 1.25; females, 3.69 ± 1.05 (*p* < 0.01, effect size = 0.38). Accordingly, item 18 ranked fifth for females and the total sample but was the eighth ranked item for males.

**Fig. 2:**
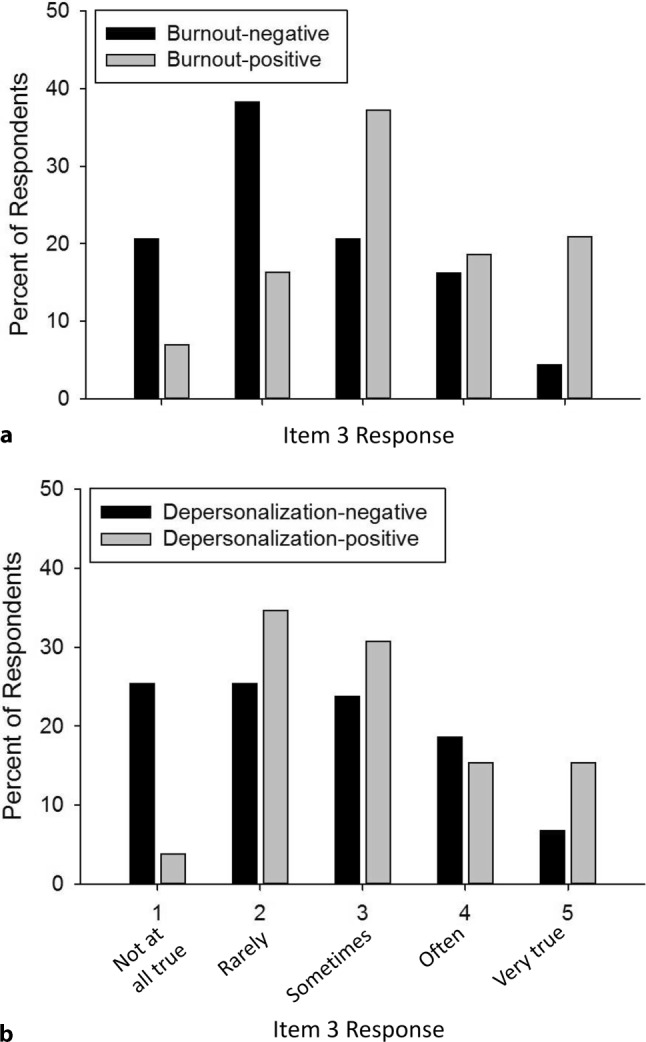
Distributions of responses to CIPS item 3, avoiding and dreading evaluations, in students responding affirmatively for burnout (**a**) or depersonalization (**b**). The mean score (± SD) was 2.56 ± 1.13 for burnout-negative (*n* = 68), 3.30 ± 1.17 for burnout positive (*n* = 43) and 2.80 ± 1.28 for depersonalization-negative (*n* = 59) and 3.17 ± 1.18 for depersonalization-positive (*n* = 52). Students positive for burnout or depersonalization endorsed higher frequency of CIPS item 3 (*p* < 0.01 and *p* < 0.05, respectively) by chi-square and Mantel-Haenszel chi-square tests

Analysis of the effects of burnout and depersonalization indicated that rating of item 3 (fear and avoidance of evaluation), which ranked 18th among CIPS items for the total sample, was higher for students who responded affirmatively for either burnout (3.30 ± 1.17 for burnout-positive v. 2.56 ± 1.13 for burnout-negative, *p* < 0.01) or depersonalization (3.17 ± 1.18 for depersonalization-positive v. 2.80 ± 1.28 for depersonalization-negative, *p* < 0.05) (Fig. 2).

There were no significant differences in the responses to specific items between students based on self-reported under-represented minority status (data not shown).

### Correlation of individual CIPS item responses with total CIPS score in the total sample.

**Fig. 3:**
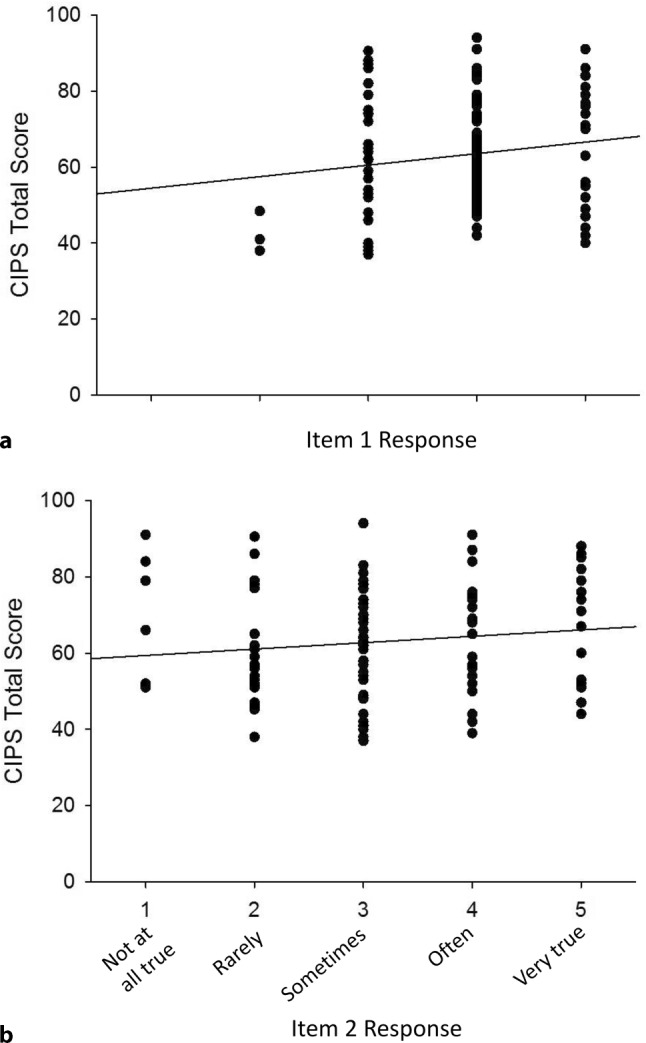
Relationship between responses to CIPS Items 1 (**a**) and 2 (**b**) and total CIPS score. Item 1 concerns unfounded fear of failure. Item 2 concerns being able to give the impression of being more competent than one actually is. For item 1, r = 0.12 by Spearman correlation. The mean score (± SD) was 3.91 ± 0.75 (*n* = 111). For item 2, r = 0.13. The mean score was 3.23 ± 1.09 (*n* = 112)

Overall, scores on individual CIPS items were moderately-to-strongly correlated with the total CIPS score (all *p* < 0.0001) with two exceptions. The exceptions were item 1 (unfounded fear of failure), where responses were notably high regardless of total CIPS score, and item 2 (able to give the impression that one is more competent than one actually is), where there was little relationship between item response and total CIPS score (Fig. 3).

## Discussion

### Impostorism in medical students

The goal of this study was to assess how impostorism manifests in medical students during the transition from the preclinical to clinical phases of training. During this period, students are notably stressed and have been shown to exhibit decreased confidence [19–21, 46]. The present findings indicate that the majority of medical students embarking on the clinical phase of their training indicate moderate-to-strong impostor feelings, a rate slightly higher than found in previous studies [11–14].

In addition to the overall general experience of impostor feelings, certain items on the CIPS were rated more strongly than others. Specifically, students reported high frequencies of having succeeded on tasks when one was afraid of failure (item 1), hesitance to reveal recognition before it is publicly announced (item 19), remembering failures rather than successes (item 7), believing themselves less capable than others (item 17), and unfounded worrying about succeeding (item 18). Thus, impostorism in this population manifests most strongly as discounting past success, comparisons to others, and fear of failure. This suggests that medical students tend of focus on performance goals and endorse a fixed mindset of intelligence, in which ability is an unchangeable entity [47].

Although responses for males and females were generally similar, females identified more strongly with item 17 (comparing self to others) and item 18 (unfounded worrying about succeeding) than males (Fig. 1). These differences are consistent with findings in undergraduates where female impostors tended to have higher grade point averages and spend more time on academics than males [48, 49]. Females have also been found to put higher importance on others’ opinions about them [47]. Individuals with low confidence were also more likely to respond to challenges with a “helplessness response” and interpret performance difficulties as evidence of a lack of ability [50, 51]. These gender differences may also result from male impostors tending to react more negatively than females when given negative feedback or when held to a high level of accountability [52]. In addition, male and female undergraduates adopted different goal achievement patterns in response to impostor feelings such that males with strong imposter feelings sought to avoid failure, whereas females focused on comparison to norms [50], consistent with the higher rating by females of comparing self to others found in this study.

Although rating of item 3, which concerns fear and avoidance of evaluation, was relatively low for medical students overall, it was more strongly endorsed by students reporting burnout or depersonalization (Fig. 2). The present findings do not establish a causal relationship for these factors. However, the significantly higher rating of this item by students positive for either burnout or depersonalization suggests that students strongly identifying with these items may have more serious wellness concerns than students who do not.

Students’ rating was notably low for item 9, which describes a lack of confidence in past successes. Similarly, items that overtly ascribe success to luck (items 5 and 11) were not highly rated. This suggests that medical students believe their prior accomplishments reflect their abilities, but question whether their abilities are sufficient for the challenges of their medical training.

Impostorism is also a concern for under-represented minorities, and incidence of impostor phenomenon has been shown to vary between undergraduates of various ethnic groups [53, 54]. In this study, however, no significant differences in the rating of CIPS items were detected between individuals self-identifying as an under-represented minority and those who did not. This failure to detect differences may have resulted from lack of power, with only 23% of the sample identifying as an under-represented minority student.

### Correlation of CIPS items with the CIPS total score in medical students

Correlation of responses to individual instrument items with the total score has been used as a measure of instrument and item validity and to assess the predictive potential of each item, but can vary depending on the demographics of the study population [32–36]. In this study, ratings of 18 of the 20 items on the CIPS had moderate-to-strong correlation with total CIPS score (Spearman’s *r* = 0.40–0.80) (Tab. 1). Consistent with previous studies [33–35], the exceptions were items 1 (succeeded on tasks when one was afraid of failure) and 2 (able to appear more competent than one actually is) where responses correlated poorly with the total CIPS score (Fig. 3). In fact, the correlations for these items were even poorer than in the prior studies (Spearman’s *r* = 0.12 and 0.11, respectively). Item 1, however, was notable in its strong rating by the third-year medical students. Factor analysis by Chrisman et al. [35] indicated that item 1 represented a fourth factor assessed by the CIPS, in addition to fake, discount, and luck. The robust response to this item describing unfounded fear of failure, even by students not reporting other prominent impostor feelings, likely reflects the challenges these students are currently facing, which are more complex and with higher stakes than those encountered by the undergraduate students or other young adults most commonly used as subjects. This suggests that item 1 could be a useful indicator of general medical student wellness status, if not impostorism. Such a relationship must be established in future studies.

### Implications for prevention and mitigation of impostorism

Recommendations for supporting those with impostor phenomenon include supportive therapeutic relationships, cognitive therapy modalities, and group therapy to help impostors see themselves more realistically and lessen dependence on external approval [55, 56]. Studies in undergraduates emphasize the importance of addressing any associated anxiety, depression, and perfectionism, and then focusing on self-control, self-esteem, adaptive goal setting, and study skills [5, 57]. Studies in older individuals suggest the value of individual coaching and relationships with mentors [6, 58]. In addition, adopting a growth mindset, in which intelligence is viewed as a developable attribute, can aid a shifting from performance goals and garnering positive evaluations to learning goals that focus on improvement and the development of skills [47, 51]. The present findings suggest that for medical students who do not tend to doubt their past accomplishments or attribute their successes to luck, these interventions should focus on mitigating the imposter beliefs of unfounded fear of failure, remembering failures rather than successes, believing themselves less capable than others, and unfounded worrying.

For female students, who were found to strongly identify with comparing themselves to others and worrying about succeeding, interventions could focus specifically on mitigation of those impostor feelings. Additional recommendations, derived from a study of female graduate students, included developing self-reliance, decreasing narcissistic expectations, and addressing deficits in self-worth [59]. Relationships with mentors, women in leadership positions, and supportive romantic partners can be beneficial [58]. Interventions raising awareness of gender bias and stigma have also been recommended for females with impostorism [49, 59].

### Limitations

The use of self-reported data from a specific time-point in training at a single medical school may limit the generalizability of the findings. Although all respondents had completed one clinical rotation, it was not possible to control for rotation or differences between the experiences in those rotations. Students who volunteered to participate in the study may have different characteristics from those who declined. The number of under-represented minorities in the total population, as well as among the respondents, was small and potentially insufficient to elucidate differences between groups. The observational nature of the study does not allow the determination of causal relationships.

## Conclusions

Medical education is demanding and the transition into the clinics is a particularly challenging phase of training. Accordingly, despite their demonstrated abilities, medical students are likely to be taxed in ways that they have not experienced previously in their academic careers, leading to feelings of impostorism. The present findings show that the specific aspects of impostorism that are strongly rated by medical students include unfounded fear of failure, hesitance to share recognition before it is announced, remembering failures rather than successes, believing themselves less capable than others, and unfounded worrying about succeeding. For females, comparing themselves to others and worrying were particularly pronounced. In contrast, doubt in past accomplishments and attribution of accomplishments to luck was not prominent for these students. In conjunction with other recommendations for mitigation of impostorism, these findings may inform the development of preventative and remedial interventions specifically tailored to foster wellness in medical students as they negotiate the transition from the preclinical to clinical phases of their training.

## Caption Electronic Supplementary Material

Supplemental Table 1. Demographic parameters
