# Supplementary material for: Impostorism in third-year medical students: an item analysis using the Clance impostor phenomenon scale
Source: Perspect Med Educ. 2020 Feb 6;9(2):83–91. doi: 10.1007/s40037-020-00562-8 (PMC7138782; doi:10.1007/s40037-020-00562-8)
Supplement: Supplementary file 1 — Supplemental Table 1. Demographic parameters [file 40037_2020_562_MOESM1_ESM.docx]

**Supplemental Table 1. Demographic parameters**

- - 1. I consent to participate in this study yes/no
    2. Age
    3. Race white/African-American/Native American/Asian/ Other
    4. Hispanic yes/no
    5. Gender male/female
    6. Under-represented minority (race/ethnicity) yes/no
    7. Under-represented minority (economic) yes/no
    8. Marital status Single/Married or Committed relationship
    9. Children yes/no
    10. Prior career outside of medicine > 2 years yes/no
    11. Desired Specialty after medical school
    12. M3 rotations completed
    13. If “other”, which
    14. M3 Current rotation
    15. If “other”, which
    16. Undergraduate GPA
    17. Composite MCAT score
    18. M1 GPA
    19. M1-M2 GPA
    20. Step 1 score
    21. Estimated average M1-M2 exam score
    22. Average shelf exam score
    23. I applied to medical school more than once yes/no
    24. I was wait-listed prior to admission to medical school yes/no
    25. Campus for M1-M2 XX/ XXXXX/ XXX
    26. Campus for M3-M4 XX/ XXXXX/ XXX
    27. Did you participation in Dr. XXXXX’s wellness program yes/no
    28. If yes, did you complete personality assessments as part of that project yes/no
